# Collagen-Based Nanofibers for Skin Regeneration and Wound Dressing Applications

**DOI:** 10.3390/polym13244368

**Published:** 2021-12-13

**Authors:** Zintle Mbese, Sibusiso Alven, Blessing Atim Aderibigbe

**Affiliations:** Department of Chemistry, University of Fort Hare, Alice 5700, South Africa; 201208394@ufh.ac.za (Z.M.); 201214199@ufh.ac.za (S.A.)

**Keywords:** biopolymers, wound healing, collagen, antibacterial agents, skin regeneration, nanofibres

## Abstract

Skin regeneration after an injury is very vital, but this process can be impeded by several factors. Regenerative medicine is a developing biomedical field with the potential to decrease the need for an organ transplant. Wound management is challenging, particularly for chronic injuries, despite the availability of various types of wound dressing scaffolds in the market. Some of the wound dressings that are in clinical practice have various drawbacks such as poor antibacterial and antioxidant efficacy, poor mechanical properties, inability to absorb excess wound exudates, require frequent change of dressing and fails to offer a suitable moist environment to accelerate the wound healing process. Collagen is a biopolymer and a major constituent of the extracellular matrix (ECM), making it an interesting polymer for the development of wound dressings. Collagen-based nanofibers have demonstrated interesting properties that are advantageous both in the arena of skin regeneration and wound dressings, such as low antigenicity, good biocompatibility, hemostatic properties, capability to promote cellular proliferation and adhesion, and non-toxicity. Hence, this review will discuss the outcomes of collagen-based nanofibers reported from the series of preclinical trials of skin regeneration and wound healing.

## 1. Introduction

The increasing global population demands many biomedical implants every year to repair lost tissues [1]. Different factors can disrupt the normal functioning of human tissues/organs including the bone, cartilage, nerves, and skin [2]. Conventional tissue replacements (e.g., allografts and autografts) have resulted in various problems (e.g., risk of infections and immune responses) that do not satisfy high-performance demands in patients [3]. Subsequently, regenerative medicine is an interdisciplinary field involving the application of engineering and life science toward the advancement of biological replacements that provide, improve, or restore tissue roles [4]. However, the various materials that are used in this field still require some improvements. The slow wound healing process is a major challenge in the arena of wound care. The factors that usually result in retarded wound healing process include bacterial invasion, underlying physiological conditions, malnutrition, prolonged bed rest, aging, etc. [5,6]. Most of the presently used wound dressings display some drawbacks, such as poor anti-inflammatory and antimicrobial effects, inability to absorb excess wound exudates, require frequent change of wound dressing, and poor mechanical properties [7,8].

**Figure 1:**
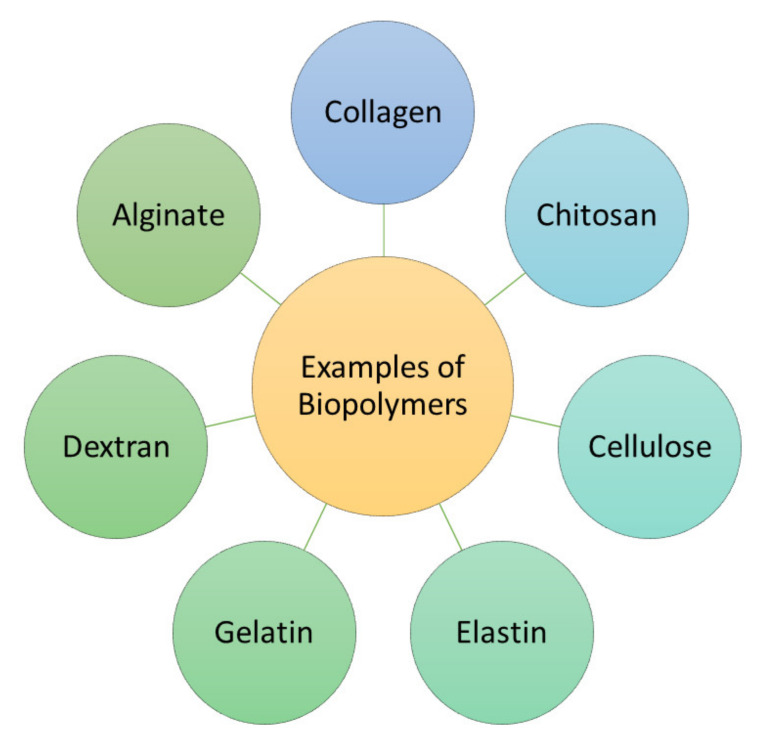
Examples of some biopolymers that are used in tissue regeneration and wound dressings.

Biopolymers such as collagen, elastin, gelatin, alginate, dextran, chitosan, and cellulose, (Figure 1) have attracted great attention from biomedical researchers in regenerative medicine and wound management because of their unique features [9]. The properties of biopolymers that make them useful in these fields include good biodegradability, excellent biocompatibility, non-toxicity, non-antigenicity and non-immunogenicity, and ready availability [10,11,12]. Some biopolymers, including chitosan, demonstrate good antibacterial activity that can be useful for the fabrication of scaffolds for the treatment of bacterial-infected injuries [13]. The common limitation of biopolymers is their poor mechanical properties, which can be overcome by combining them with synthetic polymers such as poly (ɛ-caprolactone) (PCL), polyglycolic acid (PGA), and polylactic acid (PLA), that display excellent mechanical properties [14]. Another strategy that has been employed to improve the mechanical performance of biopolymer-based materials is the use of cross-linkers [15]. The polymer of amino acids based materials that suffer from poor antimicrobial efficacy (e.g., gelatin) can be loaded with various drugs to improve their biological activities [16,17,18]. Collagen is one of the most attractive polymers useful in the field of wound care and regenerative medicine (Figure 1).

Collagen is the major constituent of the extracellular matrix (ECM) of several delicate tissues. It is found in a proportion of about 70 to 80% at the skin level, described by all dermal dry matters. The interactions between collagen and cells are essential during the process of wound healing because collagen promotes the maintenance and differentiation of cellular phenotypes [19]. Combining the properties of collagen and nanofibers (such as superior surface-area-to-volume ratio, high porosity, improved mechanical properties, excellent capacity to deliver bioactive agents) can promote skin regeneration and enhance the wound healing process [20]. Hence, this review will focus on the outcomes of collagen-based nanofibers reported in vitro and in vivo experiments in skin regeneration and wound healing.

## 2. Phases of Wound Healing

**Figure 2:**
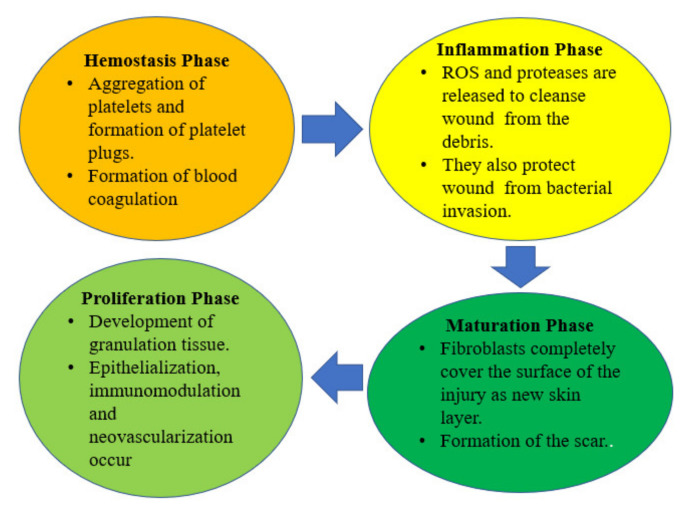
Consecutive phases of the wound healing process.

Understanding the wound healing process is an important aspect of the field of skin regeneration and wound management. Wound healing and skin regeneration are generally explained as a complex process that leads to the repair of skin tissue architecture and function to its normal state, after a disruption to the skin [21,22,23]. There are four consecutive phases of the wound healing process: hemostasis, inflammation, proliferation, and remodeling/ maturation phase (Figure 2) [24]. Hemostasis is the initial phase of wound healing and it takes place after an injury to stop excessive bleeding through a process known as vasoconstriction [25]. Primary and secondary hemostasis take place through two mechanistically and simultaneous entangled pathways. In primary hemostasis, the aggregation of platelets and the formation of platelet plugs are prompted by collagen exposure inside the subendothelial matrix [26]. In secondary hemostasis, the clotting cascade activation occurs in which soluble fibrinogen is changed to insoluble strands resulting in the formation of a fibrin network [26]. The combination of the platelet plugs and the fibrin network make up the thrombus, which stops the bleeding of the wound, releases growth factors (GFs), and a provisional scaffold for infiltrating cells essential for wound recovery [27].

The second stage of wound healing is known as the inflammation phase that usually takes place concurrently with the hemostasis phase [28,29]. The phagocytic cells release reactive oxygen species (ROS) and proteases that are vital for the cleansing of the injury from the debris [30]. It also protects the wound against bacterial invasion [30]. The blood monocytes differentiate into tissue macrophages at the wound bed that release GFs, cytokines engaging keratinocytes, fibroblasts, and endothelial cells to restore injured blood vessels. Furthermore, the epithelial cells migrate towards the injury bed to substitute the dead cells [31].

In the proliferation phase, there is a simultaneous formation of granulation tissue or connective tissue (whereby the wound is completely enclosed with epithelium) with other wound healing progressions, including re-epithelialization, neovascularization, and immunomodulation [32]. The final stage of the wound healing mechanism is the remodeling phase that is also known as maturation. The fibroblast cells shield the injured surface as a new skin epidermis layer, and a scar is formed [33].

## 3. Properties of Collagen in Biomedical Applications

**Figure 3:**
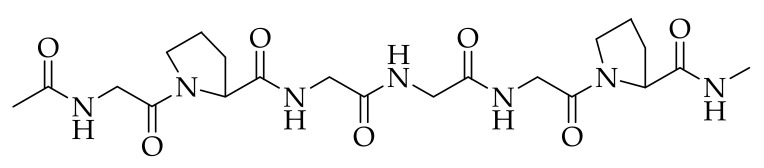
Chemical structure of collagen.

There are major qualities that must be considered when choosing a suitable material for skin regenerative and wound dressing applications. The biomaterials that were originally employed in the arena of biomedicine include ceramics and metals due to their non-immunogenic effect, but the materials such as polymers have been reported to be appropriate because of their interesting properties [34]. Biopolymers can interact with the cells, stimulating the formation of new tissues and promoting regeneration [35,36,37]. Collagen is one of the biopolymers that is often employed in skin regeneration and wound healing because of its several attractive features. The molecular structure of collagen is presented in Figure 3. Collagen is mostly obtained from porcine and cattle slaughterhouse trashes, but fishery by-products have also become a significant substitute source for collagen recently [38]. Among the 29 types of collagens that are currently known, type I collagen is the greatest, most abundant, and can be obtained from various mammalian connective tissues, such as the skin, cornea, and tendon [39,40]. Type I collagen shows the characteristic structure with two α1-chains and one α2-chain. The well-known derivative of collagen is gelatin, which is composed of the same repetition of amino acids arrangement as collagen of Gly-X-Y, where X and Y are proline and hydroxyproline, respectively [41].

Collagen is broadly employed in wound dressing and tissue engineering products because of its low antigenicity, good biocompatibility, hemostatic properties, capability to promote cellular proliferation and adhesion, and reduced cytotoxicity [42]. Various collagen wound dressing products are available in the market, some of them are summarized in Table 1. Nevertheless, most commercial collagen dressings suffer from some limitations. Some studies have demonstrated that collagen materials that are fabricated in the form of gels, films, or powder offer haemorrhage control [43]. Collagen-based porous scaffolds possess the ability to absorb large volumes of exudates, maintain moisture for the injury, thus promoting an enhanced wound healing process [44]. Collagen has been proven to be biocompatible both in vitro and in vivo, especially porcine- and bovine-derived collagen. Collagen derived from marine has resulted in biocompatible wound dressing materials that can be developed in the shape of nanofibers, sheets, hydrogels, sponges, membranes, and films [44].

Collagen-based materials that are used as porous scaffolds for the migration of cells also offer mechanical and structural support and promote the development of new tissues [45]. The collagen biomatrix imitates the natural ECM collagen and stabilizes the cellular and vascular constituents in the injury by decreasing matrix metalloproteinases (MMP) levels that are characteristically imbalanced in chronic lesions while offering structural support for the repair of tissues [46]. The modes in which the collagen wound dressing enhances the wound healing process include the capacity to connect to the GFs, control functions of the cells, enable intracellular transmission, and act as a physical structure to help tissue restoration in both chronic and acute injuries [47]. In wound healing, collagen plays a significant role in controlling inflammatory response to injury followed by repairs. It also influences the protein synthesis in the ECM, the release of growth factors and inflammatory cytokines, and the remodeling of the ECM [46,47]. Polymer-based materials are formulated by combining collagen with other polymers such as poly (ε-caprolactone) (PCL), poly (lactide-co-glycolide) (PLGA), poly (ethylene glycol) (PEG), polyglycolide (PGA), polylactic acid (PLA), and polyvinyl alcohol (PVA) [48]. These materials are also used as carrier matrices useful for accelerated wound healing mechanisms in skin injury. These materials are regularly utilized as drug delivery systems for antibiotics, GFs, essential oils, nutrients, and vitamins to further improve their wound healing activity [49,50,51].

Nanofibers formulated from various techniques, especially the electrospinning method, are often utilized as drug delivery systems making them ideal wound dressings and skin regeneration scaffolds.

**Table 1:** Summary of commercially available collagen-based wound dressings.

Form of Dressing	Composition	Product Name	Advantage	Limitations	Wounds Suitable for	Ref
Gel	Collagen	CelleraRX	Maintain moisture for wound bed	Bovine sources, and require secondary wound dressing	Partial and Full-thickness injuries including traumatic wounds, surgical wounds, diabetic ulcers, and burns	[52]
Gel	Collagen Polypeptides	Stimulen	Provide moisture for wound bed	Bovine source, and require secondary wound dressing	Full- and partial-thickness wounds including pressure ulcers, partial-thickness burns, abrasions	[53]
Pad	Collagen fleece, gentamicin salts	Septocoll E	Activate platelets	skin responses	Full and partial thickness injuries including infected wounds, and bleeding lesion	[54]
Pad	Collagen, carboxymethylcellulose, sodium alginate, AgCl	ColActive Plus Ag	Hinders the function of MMPs	Bovine sources, require secondary wound dressing	Full and partial thickness wounds including burns, dehisced surgical incisions abrasions, diabetic, venous, or pressure ulcers	[55]
Pad	Collagen and Ca alginate	Fibracol Plus	Maintain moisture for wound bed	Require secondary wound dressing	Full and partial thickness wounds including burns, dehisced surgical incisions abrasions, diabetic, venous, or pressure ulcers	[56]
Pad	Bovine collagen, and Manuka Honey	Puracol	No extra debridement Required	Bovine source and expensive.	Full and partial thickness injuries including dehisced surgical incisions abrasions, burns, diabetic, venous, or pressure ulcers	[57]
Pad	Type I equine Collagen	Biopad	Free from collagen degradation products	Equine source, time consuming, high cost	Full and partial thickness wounds including dehisced surgical incisions abrasions, diabetic, venous, or pressure ulcers	[58]
Pad	Bovine collagen and oxidized cellulose	Promogran	Hemostatic activity	Bovine source, not to be utilized in third-degree burns.	Full and partial thickness wounds including abrasions, bleeding wounds, venous or diabetic ulcers, pressure wounds	[59]
Powder	Collagen	Catrix	Decrease bleeding, Biodegradable	Bovine source, require secondary wound dressing	Full and Partial-thickness wounds including cuts, abrasions, irritations, pressure, diabetic ulcers radiation dermatitis, burns	[60]
Membrane	porcine dermal collagen, nylon, silicon	Biobrane	Flexibility	Bovine source, require secondary wound dressing	Partial-thickness burn wounds	[61]
Cellular matrix	Collagen, polycarbonate membrane	Apligraf	Resorbable	Not suitable for infected injuries, bovine source, and expensive.	Full and partial thickness injuries including venous leg ulcers, diabetic foot ulcers	[62]
Cellular matrix	Type I collagen	Orcel	Full resorbable	Not suitable for infected wounds, bovine source, high cost	Full-thickness injuries including burns	[63]

## 4. Collagen-Based Nanofibers in Skin Regeneration and Wound Dressing

**Figure 4:**
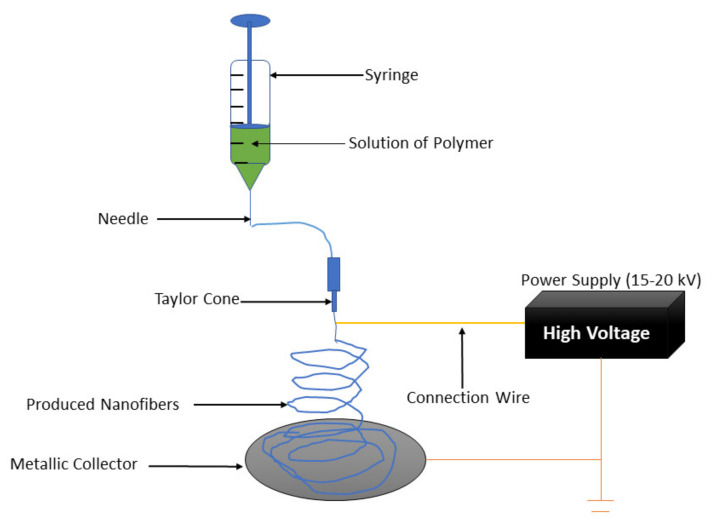
Electrospinning setup.

Wound care is gradually becoming a major public health concern globally. Most wound dressings are not effective in promoting skin regeneration. Hence, several studies are currently focused on the development of effective and novel dressing materials that can advance the wound healing process [64]. Collagen-based materials can be formulated in the form of nanofibrous scaffolds using various techniques (e.g., melt-blowing, self-assembly, template synthesis, phase separation, electrospinning, etc.) [65]. Among them all, the electrospinning technique is the most employed method in the preparation of nanofibers because of its cost-effectiveness and simplicity [66,67]. The electrospinning apparatus is composed of three parts: voltage system, spinneret system, and collecting system (Figure 4) [68,69]. The electrospinning technique utilizes high voltage electric fields to create nanofibers with diameters in various nanometers or micrometres. The spinneret system assists in the production of the near-field, and the coaxial electrospinning development has been advanced to deposit nanofibers in a controllable, direct, and continuous manner [70]. The electrospun nanofibers that possess high porosity and large surface-to-volume ratio have been beneficial in several areas, particularly in biomedical applications such as tissue regeneration, wound healing, and drug delivery systems [71,72,73,74,75]. Nanofibers are beneficial in regenerative medicine and wound healing because of their capability to imitate the ECM and stimulate the proliferation and migration of cells [76,77,78]. The features of electrospun nanofibers that make them useful in the field of wound healing are high porosity, good gaseous permeation, good cellular adhesion, good swelling capacity, and the ability to offer moisture for the acceleration of skin regeneration and wound healing process [79,80].

### 4.1. Advantages and Disadvantages of the Electrospun for Skin Regeneration and Wound Dressing

The electrospinning method allows the production of interconnected networks from fibers of nano-scale diameter and they are similar to the native structure of the natural ECM, hence they promote the normal functions of the cells, such as proliferation [64]. The flexibility and easy combination of drugs and other bioactive molecules, such as nanoparticles, antimicrobials, growth factors, and anti-inflammatory agents into the nanofibers is another significant advantage of the electrospinning method [81]. Electrospun wound dressings can provide flexibility and patient compliance. Wound dressings fabricated by electrospinning of biodegradable polymers improve patient comfort and compliance with no need for frequent changing of the dressing [82]. The biodegradable electrospun wound dressings also induce healing and enhance cell growth due to their high compatibility with tissues and blood. The degradation rate of the wound dressings can be tuned with the rate of tissue regeneration. Therefore, the aforementioned advantages make electrospun nanofibers promising materials for enhanced skin regeneration and wound healing [81,82]. However, the use of organic solvents and the limited control of pore structures is a limitation [83]. Although electrospun fibers frequently have high porosity, it is difficult to control due to dependence on the fiber diameter. Moreover, it might also limit cell penetration into the scaffold in some cases [84]. The higher voltage of electrospun might lead to more solution deposition. Thus, the properties of both the solution and the process parameters should be considered during the optimization of the electrospinning process [85,86].

### 4.2. Application of Electrospun Collagen-Based Nanofibers in Skin Regeneration and Wound Dressing

Electrospinning is a flexible and simple technique used to formulate fibers with diameters that range from micrometers to a nanometer. The polymers that have been electrospun are more than 200. The electrospinning technique has become one of the most prevalent scaffold fabrications to make nanofiber mesh for tissue engineering applications [87]. Collagen is the most abundant ECM protein in the human body; it has been electrospun to fabricate biomimetic scaffolds that imitate the architecture of native human tissues. Electrospun collagen nanofiber mesh has a high surface area to volume ratio, porosity, tunable diameter and tissue formation and also excellent biological activity to regulate cell function [87]. Currently, a lot of attention has been on fabricating biopolymer-based nanofibrous structures through the electrospinning process. The electrospinning technique is known for its low-cost and tunable method for generating ultra-fine fibers with some exceptional properties [64]. Owing to flexibility in choosing the raw materials and the possibility to tune the ultimate properties, the electrospinning method has been broadly employed for biomedical materials such as wound dressings, tissue engineering scaffolds and drug delivery systems [64]. Electrospun nanofibers can influence and interact with the damaged tissue and its biological environment according to their physical and chemical characteristics, as well as through additional linked bioactive molecules [88]. The use of crosslinkers on electrospun collagen may lead to appropriate scaffold stability and resistance to degradation in vitro and in vivo [89]. Nevertheless, more improvements in terms of their mechanical properties, the optimization of biological response, and reductions in the degradation rate are needed. Thus, several methods have been explored to combine collagen with other synthetic or natural polymers or additives through co-electrospinning, blending, and electrospinning alternating layers of the constituents or coating the electrospun fibers [89].

Deng et al. electrospun type I collagen to produce scaffolds that are similar to the native ECM within the dermis [90]. They are frequently used in skin regenerative medicine and wound regeneration. In terms of in situ crosslinked collagen-chitosan nanofibers, they have been used to improve epithelialization and angiogenesis in a rat scald model [90]. Some studies have reported the efficacy of collagen nanofibrous scaffolds in proliferation, normal human keratinocyte attachment and early-stage wound healing. A microscopic observation using identical full-thickness rectangular back injuries using a Sprague–Dawley rat as an animal model revealed early-stage wound healing in the collagen nanofiber scaffold cluster that was quicker than in the control cluster [91]. The wound surface of the control cluster was enclosed with fibrous tissue debris, along with a layer with a dense infiltration of leukocytes and an accumulation of proliferating fibroblasts. In contrast, in the collagen nanofiber scaffolds cluster, there were no surface tissue debris and fibroblast proliferation, indicating the effectiveness of collagen nanofibrous scaffolds in enhancing early-stage wound healing [91]. Pilehvar-Soltanahmadi et al. reported electrospun gelatin scaffolds as promising scaffolds for wound healing applications. Though gelatin has poor mechanical strength, it is used in combination with other materials to improve the mechanical and biological properties [91]. Electrospun collagen is a desired material for tissue engineering due to its biocompatibility and architectural versatility. The changes in the structure that happen through processing may contribute to high degradation rates that are not appropriate for numerous biomedical applications [92]. Electrospun collagen scaffolds quickly degrade in aqueous environments. Physical and chemical crosslinking of electrospun collagen improved both the mechanical properties and scaffold stability [92]. Augustine et al. developed electrospun scaffolds with antibacterial activity to inhibit wound infections [93]. The antibacterial nanofibers are commonly fabricated by incorporating antibacterial agents during electrospinning. Diverse antimicrobial agents such as metallic, nanoparticles, antibiotics, and natural extracts derived products have been loaded into electrospun nanofibers to improve their antibacterial activities. Metallic nanomaterials such as AgNPs are known as effective agents for the management of wound infections. Nanoscale particles with a high surface to volume ratio promote the antibacterial activity of electrospun wound dressings [93,94].

### 4.3. In Vivo and In Vitro Studies of Collagen-Based Nanofibers

#### 4.3.1. Plain Collagen-Based Nanofibers

Nanofibers can be loaded with therapeutic agents to enhance their biological outcomes, but in some cases, they are used as plain nanofibers without the incorporation of bioactive agents. Plain nanofibers are safe because the incorporation of bioactive agents can result in toxic reactions [95]. Collagen-based nanofibers have been reported as suitable scaffolds for skin regeneration and wound dressings. Zhou et al. formulated electrospun nanofibers for wound healing application from fish (tilapia) type I collagen [96]. The scanning electron microscope (SEM) results of collagen nanofibers displayed smooth fibers with a diameter of about 310 ± 117 nm, biomimicking the topography of the natural ECM. These nanofibers possessed good mechanical properties with a tensile strength of 6.72 ± 0.44 MPa, suitable for human skin. The in vitro studies showed that the human keratinocytes (HaCaTs) were firmly attached to the collagen nanofibers with a high proliferation rate in the first 24 h of incubation. After 5 days, the proliferation rate was further increased to 114%, indicating that these nanofibers significantly promoted cellular proliferation and adhesion with excellent cytocompatibility. The in vivo experiments utilizing Sprague–Dawley (SD) rat models further showed that the created dorsal full-thickness skin wounds were fully covered with continuous epidermis layer at day 14 when treated with collagen-based nanofibers. Those treated with control (wound dressing called Kaltostat) were not yet completely covered with the epidermis, suggesting that these nanofibers accelerated the wound healing process [96]. These results showed that electrospun nanofibers that are based on fish (tilapia) collagen can significantly improve the healing of chronic wounds. Collagen, as well as the structure of nanofibers, imitate natural ECM. A similar study was conducted by Zhou et al. that demonstrated electrospun collagen-based nanofibers’ capability to significantly accelerate skin regeneration of full-thickness wound in the SD rat model, indicating their potential use in biomedical applications [96].

Fu and Wang fabricated pristine collagen- poly (ε-caprolactone) (PCL) hybrid nanofibers using an electrospinning method [97]. They studied the effect of human adipose stromal cells (hASCs) on wound healing. The in vitro studies revealed high proliferation, migration, and adhesion rate of hASCs with good biosynthesis of collagen when cultured with the hybrid nanofibers. The hybrid nanofibers were formulated from collagen (8% w/v), and PCL solution (8% w/v) at a volume ratio of 1:3 and resulted in high cell spreading and adhesion of hASCs, showing that the high amount of PCL favoured cellular attachment and proliferation [97]. These results confirmed that these electrospun collagen-PCL hybrid nanofibers can accelerate the wound healing process. Sobhanian et al. prepared nanofibrous rat tail collagen-grafted alginate/gelatin/PVA scaffolds as potential wound dressing materials [98]. The Fourier transform infrared spectroscopy (FT-IR) confirmed the successful preparation of electrospun hybrid nanofibers. The Water Vapor Transmission Rate (WVTR) study of nanofibers was approximately 1575.72 gm^−2^ day^−1,^ appropriate for maintaining a moist environment for the wound healing process. The indirect 3-(4,5-dimethylthiazol-2-yl)-2,5-diphenyl tetrazolium bromide (MTT) assay and direct MTT assay using European Collection of Authenticated Cell Cultures (ECACC 85011425) known as L929, and human fibroblast cells, respectively, resulted in high cell viability when seeded with the nanofibrous scaffolds, confirming the non-toxicity of the scaffolds that might be due to the high amount of PVA in the electrospun hybrid nanofibers, which were fabricated from collagen solution (1% *w*/*v*), and PVA solution (5% *w*/*v*), and gelatin solution (2% *w*/*v*). [98]. These cytocompatibility results further showed that these nanofibers promote high cell growth and attachment that is crucial during wound healing.

Rho et al. synthesized type I collagen-based nanofibers utilizing the electrospinning method for wound healing [99]. The porosity studies of electrospun collagen nanofibers using mercury porosimetry displayed high porosity that ranged between 70.83% and 89.21%, indicating their suitability for treating injuries due to its ability to allow gaseous exchange and nutrient diffusion [99]. The in vivo studies revealed a similar wound healing effect of collagen nanofibers and reference (gauze) on SD rat full-thickness wounds on day 7 and day 10; nevertheless, the histological assessment showed that early-stage wound healing for wounds dressed by the collagen nanofibers was faster than those dressed treated with the gauze [99]. These electrospun nanofibers showed their potency in wound healing and skin regeneration. Powell et al. fabricated collagen-based nanofibers as potential wound dressing scaffolds [100]. The wound closure experiments of nanofibers showed good wound recovery activity on mice wound models with the formation of a uniformly dried epidermis after 14 days [100]. The histological examination displayed excellent skin cell migration, revealing their ability to promote skin regeneration [100]. The collagen-hyaluronic acid nanofibers (Gelfix^®^ spray) studied by Elibol et al. revealed the good healing effect of the nanofibers on vocal cord injury of Zealand white rabbits [101]. In vivo studies on the right vocal cords of New Zealand white rabbits were performed by administering 1.08 mg/75 mL of the topical HA-collagen nanofiber (Gelfx^®^ spray) for a period of 3 days, revealed collagen bundles in the granulation tissue [101].

Deng et al. prepared recombinant human collagen peptide/chitosan hybrid nanofibers using the electrospinning technique for application in wound management [90]. The SEM results of the electrospun nanofibers displayed uniform, random distribution and showed bead-free, smooth structures with mean fiber diameters of approximately 168 ± 58 nm. The fibers nanometer was found to be in a range appropriate for skin regeneration because they imitate natural ECM [90]. The WVTR of the nanofibers was 2693 g m^−2^ d^−1^, revealing their capacity to provide appropriate moisture for highly exuding injuries without causing excessive dehydration. The mechanical characterization displayed a tensile strength of 8.09 MPa, and Young’s modulus 38.16 MPa, and elongation break of 32.58%, respectively, which are at the range of human skin. The cytotoxicity studies using the MTT test showed high cell viability of HUVEC and NIH 3T3 cells when cultured with collagen nanofibers for 7 days, demonstrating good cytocompatibility and non-toxicity [90]. The in vivo examinations using a deep second-degree scald model on the SD rat dorsal skin demonstrated accelerated scald recovery, and this was significant on the 11th and 14th days [90]. Yu et al. synthesized electrospun collagen/chitosan hybrid nanofibers for skin defect repair [102]. The in vitro experiments showed that the skin keratinocytes and fibroblasts extracted from SD rat skin were well proliferated on and within the hybrid nanofibers, indicating excellent biocompatibility. These collagen nanofibers are promising scaffolds for skin regeneration [102].

Vigneswari et al. formulated electrospun collagen peptides/P(3-hydroxybutyrate-co-4-hydroxybutyrate) hybrid nanofibers for injury care [103]. The FTIR analysis confirmed the successful preparation of electrospun hybrid nanofibers. The cytocompatibility experiments showed an accelerated cell growth of the L929 fibroblast cells over 3 days when cultured with the nanofibers, indicating good cytocompatibility and non-toxicity, which are properties of an ideal wound dressing. The in vivo experiments using the hybrid nanofibers indicated a significant and accelerated wound healing process with 98% wound closure (almost completely healed) on 14 days when compared with P(3-hydroxybutyrate-co-4-hydroxybutyrate) (88% wound contraction) and control (gauze) that resulted in only 63% wound closure [103]. These hybrid nanofibers exhibited very important properties of an ideal wound dressing that include non-toxicity, excellent biocompatibility, and a faster wound healing mechanism, making them suitable candidates for skin regeneration and wound recovery. Ma et al., fabricated collagen-PLGA nanofibers using the electrospinning technique [104]. The Transmission Electron Microscopy (TEM) and SEM images displayed nanometer scale and beadless morphology of the hybrid nanofibers, biomimicking ECM [104]. Contact angle studies showed a decreased hydrophobic nature of the collagen-based hybrid nanofibers than the plain PLGA nanofibers and revealed its potential to induce cell attachment and growth that can result in a good wound healing process [104]. Huang et al. designed collagen/PCL hybrid nanofibers employing the electrospinning method for wound healing application. The SEM results of the hybrid nanofibers showed a relatively smooth surface with a fiber diameter of about 313.33 ± 69.52 nm, mimicking natural ECM. The in vitro studies utilizing both MTT assay and DNA assay was a time-dependent increase of the cell number, which indicates the excellent biocompatibility of hybrid nanofibers for the proliferation of normal human dermal fibroblasts (NHDFs) [105]. The excellent cytocompatibility and high rate of cell proliferation can result in significantly improved wound healing.

Several features displayed by the plain collagen-based nanofibers make them promising scaffolds for skin regeneration and wound healing applications. The moderate WVTR that is shown by these scaffolds is a crucial feature responsible for the prevention of dehydration and accumulation of excess exudates of the wound bed, and the results indicate their appropriateness to provide a moist environment for wound healing. Their high porosity can provide good gaseous diffusion and promote high cell proliferation and adhesion, and nutrient migration, promoting accelerated wound healing. Several in vitro studies of plain nanofibers resulted in a high rate of cell growth, cell attachment, cell viability, suggesting their excellent biocompatibility and non-toxicity. The combination of collagen and other polymers (PEG, PCL, PLLA, etc.) to obtain a hybrid nanofiber that possessed excellent mechanical properties similar to the human skin. All the aforementioned factors of collagen-based nanofibers in accelerating the wound healing process, especially in vivo, makes them suitable candidates for wound dressing and skin regeneration applications. Although these scaffolds demonstrate these crucial factors, they also suffer from poor biological activities (e.g., antibacterial activity and antioxidant efficacy), which are requirements for wound dressings that are effective for the treatment of chronic wounds. The incorporation of bioactive agents into the collagen nanofiber can be employed and used to treat chronic wounds [77].

#### 4.3.2. Collagen-Based Nanofibers Loaded with Bioactive Agents

Various bioactive agents have been incorporated into wound dressings to improve their therapeutic outcomes. These bioactive agents include antibiotics (e.g., ciprofloxacin, metronidazole, gentamicin, norfloxacin, etc.), metal-based nanoparticles (e.g., Ag and Zn nanoparticles, etc.), plant extracts (aloe vera, curcumin, etc.), growth factors, vitamins, etc. [106]. These therapeutic agents can significantly improve the biological activities of nanofibers. The ability of nanofibers to deliver drugs is one unique feature that makes them ideal for wound care [107]. The dressings that can be also utilized as drug delivery systems are called bioactive wound dressings. Several research studies revealed collagen-based nanofibers as suitable bioactive wound dressings both for skin regeneration and wound healing applications. Ghorbani et al. prepared electrospun collagen/PCL/zein hybrid nanofibers co-loaded with aloe vera and zinc oxide nanoparticles (ZnO NPs) for wound dressing application. The mechanical performance of the nanofibers includes increased tensile strength [108]. The in vitro biodegradation studies showed weight loss of over 30% of the initial weight for the Zein/PCL nanofibers with a ratio of 90:10, 42% for Zein/PCL nanofibers with a ratio of 80:20, and 54% for the Zein/PCL nanofibers with a ratio of 70:30, indicating that the high amount of PCL increased the rate of biodegradation. The cytotoxicity study of co-loaded nanofibers displayed improved fibroblast cell proliferation and attachment compared to the plain nanofibers, signifying good biocompatibility and non-toxicity. The in vitro antimicrobial studies showed that the plain nanofibers possessed no inhibition effects against *Escherichia coli* (*E.coli*) (Gram-negative) and *Staphylococcus aureus* (*S. aureus*) (Gram-positive) while the drug co-loaded hybrid nanofibers demonstrated high inhibition zones against both *E. coli* and *S. aureus* strains of bacteria [108]. The loading of bioactive agents (ZnO NPs and Aloe vera) improved biocompatibility and antibacterial activity, suggesting a synergistic effect of dual drug-loaded nanofibers, making these scaffolds ideal for wound dressings. The collagen-based nanofibers loaded with Ag NPs were formulated by Rath et al. and the nanofibers demonstrated excellent antibacterial efficacy against *S. aureus* and *Pseudomonas aeruginosa,* known wound pathogens [109]. The in vivo investigations showed an accelerated wound recovery rate of the nanofibrous mats than the pristine nanofiber mats. Histology studies also revealed accelerated collagen production, re-epithelization, and superior wound closure with collagen nanofiber mats loaded with AgNPs [109].

Khartini et al. fabricated collagen/PCL hybrid nanofibers incorporated with gentamicin sulfate for skin regeneration application [110]. The SEM images of nanofibers exhibited a randomly organized network with a mean fiber diameter of approximately 119.90 ± 21.97 nm, and incorporation of up to 3% gentamicin did not affect the morphology of nanofibers [110]. The in vitro cytotoxicity studies revealed high cell viability of human dermal fibroblast (HDF) cells when seeded with gentamicin-loaded nanofibers for 5 days, suggesting non-toxicity that can result in high cell proliferation and adhesion rate that are required for skin regeneration [110]. The drug release studies in vitro exhibited initial rapid release of about 87.5% gentamicin sulfate at the first 24 h followed by a sustained and slow drug release for 72 h. The drug release mechanism is attributed to the high amount of PCL because collagen and PCL were blended at a ratio of 1:3 (*v*/*v*) to formulate electrospun hybrid nanofibers [110]. The initial burst release of gentamicin is useful in killing the bacterial strains that are available at the wound bed and the sustained release can protect the wound from further bacterial infections. Tort et al. synthesized collagen-based nanofibers loaded with doxycycline via an electrospinning method [111]. The mechanical properties of the nanofibers were 7.65–9.46% elongation at break values and tensile strength between 2.76 and 3.47 MPa revealing similar mechanical features of human skin. The wettability studies showed that the contact angles of nanofibers were lower than 90^o^, suggesting these nanofibers possess a hydrophilic nature that can stimulate cell growth and attachment during wound healing. The drug release profile showed that doxycycline was released rapidly from the nanofibers containing 1% PCL than those nanofibers that do not contain PCL, suggesting that the presence of PCL can promote a rapid drug release mechanism. The in vitro cell culture study displayed high cell viability of HaCaT and fibroblast cell lines when incubated with doxycycline-loaded nanofibers indicating their non-toxic property [111].

Selvaraj et al. prepared collagen-silk fibroin nanofibers loaded with an antioxidant extract known as fenugreek for wound dressing application [112]. The FTIR analysis confirmed the successful preparation of fenugreek-incorporated hybrid nanofibers. The porosity studies displayed that the loading of an antioxidant agent decreased the porosity of nanofibers, but it was optimum to enhance the extracellular migration, cell adhesion, and high gaseous permeation thereby, supporting effective wound recovery. The in vitro antioxidant experiments of the hybrid nanofibers using 1,1-diphenyl-2-picrylhydrazyl (DPPH) scavenging test exhibited good antioxidant efficacy that was induced by the presence of polyphenols in the fenugreek extract and amino acids (tyrosine and tryptophan) in the silk fibroin. The in vivo studies employing the rat model revealed that the full-thickness lesions treated with fenugreek-loaded hybrid nanofibers healed faster when compared with the control (gauze) [112]. The good antioxidant activity of hybrid nanofibers is advantageous for the treatment of chronic injuries with a prolonged inflammatory phase. Yao et al. fabricated collagen/gelatin/chitosan nanofibers loaded with *Lithospermi radix* extract for wound treatment. The in vivo wound healing studies using extract-loaded nanofibers offered good wound healing rates in the rat models over a period of 14-days compared with gauze and the commercially available dressing (Comfeel^®^) [113].

Ribeiro et al. prepared collagen-based nanofibers embedded with nanophase hydroxyapatite for skin regeneration. The porosity studies exhibited outstanding porosity with high interpore-connectivity, predominantly vital to permit the transfer of exudates from the wound bed. The proliferation rates significantly decreased with the incubation time as the increasing number of HDNF cells reduced the available space in vitro [114]. Hou et al. fabricated collagen-polyamide hybrid nanofibers encapsulated with *N*-acetylcysteine (an antioxidant) for application in wound dressing [115]. The water uptake analysis of the antioxidant-loaded nanofibers was high, with good water absorption capability, revealing their capability to absorb blood, wound exudates, and necrotic tissues, which is advantageous for the prevention of microbial infections. The drug release profile of *N*-acetylcysteine from the nanofibers was sustained for two weeks. The sustained drug release pattern has the potential to prevent inflammatory reactions that contribute to delayed wound healing in chronic injuries. The in vivo experiments using SD rat model with full-thickness wounds showed that N-acetylcysteine-loaded hybrid accelerated wound healing (almost completely closed at day 14) than those dressed with plain hybrid nanofibers and polyamide-based nanofibers. The findings revealed the efficacy of loading *N*-acetylcysteine in nanofibers-based wound dressings [115].

Zhou et al. fabricated fish collagen-based nanofibers encapsulated with bioactive glass for application in skin regeneration [116]. The mechanical properties of the bioactive glass-loaded nanofibers were an excellent tensile strength of 21.87 ± 0.21 MPa in dry conditions but decreased to 4.39 ± 0.23 MPa in wet conditions. The antibacterial studies in vitro showed that the bioactive glass-loaded nanofibers significantly inhibited proliferation and adhesion of *S. aureus*, while the pristine fish collagen nanofibers did not display any significant bactericidal effect. The MTT assay showed that these nanofibers promoted the proliferation and adhesion of HaCaTs that can play an essential role in the re-epithelialization of the injury bed. The wound closure results demonstrated that the skin wounds on the SD rats that were treated with bioactive glass-loaded nanofibers were the smallest at day 14. The bioactive glass-loaded nanofibers stimulated rapid re-epithelialization [116]. The ostholamide-incorporated collagen/gelatin/PHB fabricated by Kandhasamy et al. demonstrated outstanding wound healing in vivo in full-thickness wound model in Wistar rats with good antibacterial activity against *P. aeruginosa* and *S. aureus* in vitro [117]. The results showed that these nanofibers can be used for the treatment of bacterial infection wounds.

Most of the properties that are displayed by plain collagen-based nanofibers are also demonstrated by collagen nanofibers that are loaded with bioactive agents. The common factor that makes bioactive agent-loaded nanofibers superior to pristine nanofibers is their improved biological activities. In most cases, pristine collagen nanofibers do not show any significant antibacterial or antioxidant activity compared to the drug-loaded collagen nanofibers that exhibit excellent antibacterial and antioxidant activity. An initial rapid drug release profile followed by a sustained drug release profile is one important characteristic of nanofibers that contributes to their distinct biological activity. Moreover, the dual drug-loaded collagen nanofibers demonstrate superior therapeutic effects suggesting a synergistic effect. Nevertheless, the loading of a high amount of bioactive agents in electrospun collagen nanofibers can result in decreased porosity that can lead to low gaseous permeation and a low rate of cellular proliferation and adhesion. The amount of drugs to be loaded into nanofibers must be considered.

#### 4.3.3. Other Collagen-Based Nanofibrous Scaffolds

Other nanofibrous scaffolds are often utilized in tissue engineering and wound healing. These scaffolds include nanofibrous membranes, nanofibrous mats, nanofibrous sponges, etc. They display similar properties as nanofibers and are normally fabricated by the electrospinning technique. Some research reports are based on collagen-based nanofibrous with or without bioactive agents for application in skin regeneration and wound dressing. Lai et al. fabricated collagen/hyaluronic acid (HA) nanofibrous membrane incorporated with various growth factors [vascular endothelial growth factor (VEGF), basic fibroblast growth factor (bFGF), platelet-derived growth factor (PDGF), and endothelial growth factor (EGF)] [118]. The in vitro release profile of the growth factors was an initial rapid release from the collagen nanofibrous membranes followed by a slow release. The growth factors were slowly released for over 1 month after the rapid release from the nanofibrous matrix, indicating that the integrity of growth factors was preserved due to the ECM components (gelatin and HA) in nanofibrous scaffolds. Other in vitro studies showed a high rate of proliferation of HUVECs when incubated with the growth factor-loaded nanofibrous membranes for 14 days, indicating good biocompatibility and non-toxicity, which are crucial properties of an ideal wound dressing material. The in vivo examinations utilizing full-thickness wounds on diabetic SD rats displayed complete wound contraction for wounds dressed with growth factor-loaded collagen nanofibrous membranes at 4 weeks while most of the full-thickness injuries were closed at 6 weeks in plain nanofibrous membranes, nanofibrous membranes loaded with only two types of growth factors or control (some commercial dressing) [118].

Lee et al. prepared electrospun nanofibrous collagen/PLGA scaffold membranes encapsulated with glucophage for the treatment of diabetic wounds [119]. The SEM images displayed the diameters of the electrospun glucophage-loaded hybrid nanofibrous membranes of about 203 ± 41 nm which were significantly smaller than those of the plain hybrid nanofibrous membranes (254 ± 45 nm), and the porosity of the nanofibrous membranes was significantly high. The wettability experiments showed that the incorporation of glucophage significantly increased the hydrophilic nature of the collagen nanofibrous membranes that can cause high cell proliferation and attachment during the wound healing process. The in vitro drug release studies showed that the glucophage-encapsulated nanofibrous membranes released the drug for 21 days, with an initial burst in two days that might be due to high amount of PLGA (240 and 280 mg for group A and B, respectively) than collagen (120 mg for both group A and B nanofibrous membranes) in nanofibrous scaffolds. The in vivo wound healing studies employing the rat model revealed that electrospun glucophage-loaded hybrid nanofibrous membranes significantly accelerated the wound healing process (completed at day 14) of diabetic wounds than the pristine hybrid nanofibrous membranes [119]. The study conducted by Liu et al. demonstrated that 2 and 3 weeks after surgery on the rat model, the wound healing of the plain electrospun nanofibrous collagen/PLGA scaffold membrane group was faster than those treated with the commercial dressing and gauze group [120]. The nanofibrous scaffolds of PLGA and collagen were prepared at concentrations of 15% and 8% (*w*/*v*) respectively, and the in vitro studies demonstrated good cytocompatibility on human fibroblasts when incubated with nanofibrous scaffolds [120]. The berberine-loaded collagen/zein nanofibrous membranes formulated by Lin et al. demonstrated excellent wound healing activity on the SD rat wound model [121].

Chen et al. synthesized pristine electrospun collagen/chitosan/polyethylene oxide (PEO) hybrid nanofibrous membrane wound management [122]. The SEM micrographs of nanofibrous membranes confirmed the nanofibrous nature of the electrospun membranes with a fiber diameter of about 134 ± 42 nm. The in vitro cytotoxicity results from MTT assays showed good biocompatibility and the capability of the nanofibers to support cellular growth and normal functions of fibroblasts, suggesting non-toxicity. The high concentration of PEO (3.5 %*w*/*v*) in the total polymer concentrations of 5 %*w*/*v* in the nanofibrous membranes significantly led to good in vitro biocompatibility and promoted the growth and normal functions of the fibroblasts. The in vivo studies showed that the injury areas gradually decreased and reached about 5% after 21 days when covered with the nanofibrous membranes, and the nanofibrous membranes were found to be superior to the gauze and collagen sponge in stimulating the wound healing process [122]. Venugopal et al. formulated electrospun collagen-PCL nanofiber membranes for wound treatment. The nanofibrous membranes possessed good porosity which is appropriate for cells proliferation and adhesion of fibroblasts and are potential scaffolds for skin regeneration [123].

Ahmadian et al. fabricated collagen/ethylcellulose/polylactic acid nanofibrous mats loaded with Ag sulfadiazine with antimicrobial efficacy for wound treatment [124]. The FTIR results confirmed that Ag sulfadiazine was successfully loaded into the hybrid nanofibrous mats. The fabricated nanofiber mats possessed good drug release properties of Ag sulfadiazine in vitro, suitable for wound dressing applications. Furthermore, the enhanced cell proliferation and attachment of NIH 3T3 cells in the presence of the plain nanofiber mats and Ag sulfadiazine-loaded nanofiber mats in comparison with control groups (cells without any nanofibers), revealed the excellent biocompatibility and non-toxicity of Ag sulfadiazine-loaded collagen nanofiber mats, which are crucial properties of an ideal wound dressing. The in vitro antibacterial experiments using the disc diffusion method showed that the nanofibrous mats loaded with 0.75% Ag sulfadiazine possessed higher inhibition zones than mats loaded with the low amount of Ag sulfadiazine against *E. coli* and *Bacillus* (Gram-positive) bacteria [124]. The nanofibrous scaffolds demonstrated exactly the properties that are exhibited by nanofibers. The properties of nanofibrous scaffolds are excellent biocompatibility, non-toxicity, high porosity, ability to be loaded with bioactive agents, and accelerated wound healing.

## 5. Integrity of Collagen during the Fabrication of Nanofibers and Standardization of Raw Collagen

Collagen obtained from different sources differs in their physicochemical properties slightly **[125]**. Collagens obtained from frog skin, bird feet, shark skin, and sea urchin has a molecular structure that is different from those obtained from domestic animals [126,127,128]. Furthermore, their thermal property, peptide constitution, amino acid composition, and content of glycosaminoglycan are significantly different from collagen isolated from land animals [126]. Collagen is also isolated from yeast, plants, bacteria, etc., and is known as recombinant human collagen [129]. However, it is expensive and isolated in poor yield but overcomes the risk of transmission of diseases and batch-to-batch variations [130]. The commonly electrospun collagen types are collagen I-IV. II, III and IV. Several factors make electrospun collagen nanofibers superior compared to nanofibers prepared from other polymers such as their capability to mimic the native tissues, their poor immunogenicity, capability to activate the host immune response, and excellent biocompatibility. However, their shortcomings are poor mechanical properties which can be improved by cross-linking with synthetic polymers [126]. The sources of collagen affect the properties of the nanofibers. Most of the studies on collagen-based nanofibers reported the use of type I bovine skin collagen to develop electrospun collagen nanofibers. The molecular weight of collagen has been reported to influence the formation of nanofibers. Low molecular atelocollagen did not form fibers [131]. Similar findings were reported by Zeugolis et al. in which the source of collagen influenced their capability to form fibers and also the properties of the formed fibers [132]. Collagen type I isolated from human placenta used in the design of nanofibers resulted in less uniform fibers and a larger range of diameter [133]. Choosing an ideal solvent for collagen electrospinning for the formation of the fibers without compromising the integrity of collagen is crucial. Solvents such as 1,1,1,3,3,3-hexafluoro-2-propanol, acetic acid, 2,2,2-trifluoroethanol, and phosphate buffered saline/ethanol have been commonly used for the preparation of collagen nanofibers. Using 1,1,1,3,3,3-hexafluoro-2-propanol promoted collagen fiber formation but denatured it [126]. It can also result in gelatin fibers because the denatured form of collagen is gelatin which is obtained when the triple helical structure is denatured [134]. Using acetic acid resulted in fibers with a more triple helical structure when compared to using 1,1,1,3,3,3-hexafluoro-2-propanol for the development of electrospun nanofibers [135]. Using glacial acetic acid in combination with DMSO in a 93:7 ratio produced collagen nanofibers with retained features of native collagen [136].

## 6. Conclusions and Future Perspective

The collagen-based nanofibers or nanofibrous scaffolds that were studied in a series of in vitro and in vivo experiments displayed promising outcomes that are very essential in wound healing and skin regeneration. Different animal models were used for the in vivo studies. Although animal models are appropriate for studies on wound healing, the complex biological pathway in wound healing does not always reflect in animal models.

The use of small animal models also limits the type of wounds that can be investigated. Securing the wound dressings to small animals can be challenging and can also affect the results obtained. Small animal models also have differences in their anatomy and physiology. In most of the reports, the rationale for selecting the models used for the in vivo studies was not reported. Some properties of collagen-based nanofibers include high porosity, excellent gaseous diffusion, and moderate WVTR to maintain a suitably moist environment for skin regeneration and wound healing, non-toxicity, excellent biocompatibility, and capability to stimulate high cell proliferation and adhesion rate. Collagen nanofibers can be encapsulated with therapeutic agents for enhanced biological activities. Most collagen nanofiber wound dressings displayed an initial rapid release of bioactive agents followed by slow and sustained drug release, which resulted in good biological efficacy and the protection of the wound from microbial infections and oxidation reactions, demonstrating that these nanofibers can be very useful in the treatment of chronic wounds. The drug release mechanisms can be caused by the diffusion of the loaded bioactive agents from the polymeric nanofibers or biodegradation of the polymeric materials or the combination of both factors. The combination of collagen and other polymers, especially synthetic polymers, can result in an excellent mechanical performance of nanofibrous scaffolds, which is a property of an ideal wound dressing. The co-loading of antibacterial or antioxidant agents together with growth factors can lead to potential nanofibrous wound dressings that possess both good therapeutic activities and stimulate skin cell proliferation and adhesion. All the aforementioned properties reveal that more collagen-based nanofibrous scaffolds will reach clinical trials. Most commercially available collagen-based wound dressing products that are listed in Table 1 are expensive and require a secondary dressing. Wound dressings that require frequent changes result in a high cost of wound care. Using a secondary dressing also add cost to wound care. Cost-effective and ideal wound dressings that can be formulated from simple technology like the electrospinning method are urgently required.
